# Habitat features and colony characteristics influencing ant personality and its fitness consequences

**DOI:** 10.1093/beheco/araa112

**Published:** 2020-11-10

**Authors:** István Maák, Gema Trigos-Peral, Piotr Ślipiński, Irena M Grześ, Gergely Horváth, Magdalena Witek

**Affiliations:** 1 Museum and Institute of Zoology, Polish Academy of Science, Wilcza 64, Warszawa, Poland; 2 Department of Environmental Zoology, Institute of Animal Sciences, Agricultural University, Al. Mickiewicza 24/28, Kraków, Poland; 3 Behavioural Ecology Group, Department of Systematic Zoology and Ecology, ELTE Eötvös Loránd University, Egyetem tér 1–3, Budapest, Hungary

**Keywords:** aggression, behavioral syndrome, colony size, exploration, nest displacement, residual intraindividual variation

## Abstract

Several factors can influence individual and group behavioral variation that can have important fitness consequences. In this study, we tested how two habitat types (seminatural meadows and meadows invaded by *Solidago* plants) and factors like colony and worker size and nest density influence behavioral (activity, meanderness, exploration, aggression, and nest displacement) variation on different levels of the social organization of *Myrmica rubra* ants and how these might affect the colony productivity. We assumed that the factors within the two habitat types exert different selective pressures on individual and colony behavioral variation that affects colony productivity. Our results showed individual-/colony-specific expression of both mean and residual behavioral variation of the studied behavioral traits. Although habitat type did not have any direct effect, habitat-dependent factors, like colony size and nest density influenced the individual mean and residual variation of several traits. We also found personality at the individual-level and at the colony level. Exploration positively influenced the total- and worker production in both habitats. Worker aggression influenced all the productivity parameters in seminatural meadows, whereas activity had a positive effect on the worker and total production in invaded meadows. Our results suggest that habitat type, through its environmental characteristics, can affect different behavioral traits both at the individual and colony level and that those with the strongest effect on colony productivity primarily shape the personality of individuals. Our results highlight the need for complex environmental manipulations to fully understand the effects shaping behavior and reproduction in colony-living species.

## Teaser

The behavior of your family can depend on the place you live and may affect the amount of resources you gain. We studied this relationship in ants living in two environmentally different meadows. Ants living in big families and in more crowded places have to be aggressive to produce more brood. Ants living in less crowded but food-limited places have to move more to reproduce successfully. Exploration skills are beneficial in both habitats.

## INTRODUCTION

Studies on behavioral variation, including research on animal personality—nonrandom among-individual behavioral variation consistent over time and/or ecological situations (i.e., change in conditions)—have become a prominent field of behavioral ecology in the last two decades (Réale et al. 2007; Sih et al. 2012). Behavioral traits, like aggression, boldness, activity, or exploration, are frequently used by ecologists to test whether animals are repeatable and whether distinct behaviors are correlated (Sih et al. 2010). If correlated, they are referred to as “behavioral type” in the context of an individual unit of a group and “behavioral syndrome” as the property of groups, for example, populations (Sih et al. 2010). Most personality-related studies have been conducted on vertebrates, nevertheless, a substantial number of research conducted on a wide range of arthropod taxa also reported personality differences (e.g., insects: Kralj-Fišer and Schuett 2014; Planas-Sitja et al. 2015; Santostefano et al. 2017; Niemelä et al. 2019; crustaceans: Briffa 2013; Horváth et al. 2019).

Nowadays, animal behavioral studies dedicate more attention to the degree of behavioral plasticity of individuals and its adaptive significance (e.g., Dingemanse et al. 2010; Keiser et al. 2018), as individuals can show variation in their reaction to changes in the environment (behavioral plasticity; see Dingemanse et al. 2010; Westneat et al. 2011; Dingemanse and Wolf 2013; Mitchell and Biro 2017). However, even after accounting for environmentally induced intraindividual variation, a considerable residual variation remains, the so-called residual intraindividual variance in a phenotype (e.g., Westneat et al. 2013). A growing number of both theoretical and empirical studies suggest that residual intraindividual variation (hereafter: rIIV; see Biro and Adriaenssens 2013; Briffa 2013; Briffa et al. 2013), or in other words, the “rigidity” of an individual’s mean behavior in a certain environment should be considered as potentially independent components of individual behavioral strategy (Dingemanse et al. 2010; Briffa 2013; Dingemanse and Wolf 2013; Westneat et al. 2013, 2015; Mitchell et al. 2016).

In social insects, natural selection acts not only on individuals but mostly at the colony level (perceived as a reproductive unit) (Korb and Heinze 2004), thus social species are excellent for studying the effect of behavioral variation on colony fitness, as this can be measured at different levels of their organization. Moreover, similarly to workers, which are usually sterile individuals performing different tasks inside the nest, colonies also display consistent behavioral differences, showing in this way both individual and colony-level personality (Jandt et al. 2014; Jeanson and Weidenmüller 2014; Wright et al. 2019). Therefore, considering colonies of social insects, various levels of behavioral variation can be measured: intraindividual variability of workers, among-individual variation of workers within the same colony, and behavioral variation among colonies coming from the same population. However, the proximate mechanisms affecting intraindividual variability as well as its wider ecological significance are still little explored and not studied in social insects (Keiser et al. 2018).

In general, the factors influencing individual and colony personality can be ascribed to two main categories: genetic and environmental (Wright et al. 2019). Many biotic and abiotic environmental characteristics, such as, for example, colony size (Dornhaus et al. 2012), population density (Modlmeier and Foitzik 2011), nest structure (Pinter-Wollman et al. 2012), or the experience gained by certain group members (Jeanson and Weidenmüller 2014; Gordon 2016) can influence the variability of collective behaviors or the mixture of different individual behaviors in a group (Pruitt and Goodnight 2014). Moreover, age and the social and local environment (e.g., food availability, competition, predation, and climate) can influence the gene expression of individuals and determine their personality (Bengston and Jandt 2014; Wright et al. 2019). However, the main issue is to understand the mechanisms leading to individual and group differences in the behavior.

Pinter-Wollman (2012) pointed out that the personality of social groups can depend on the personalities of the individuals comprising them. Based on some new results, a direct linear link between individual and colony behavior can be found, suggesting that the colony personality can be the average personality of workers involved in a given task (Carere et al. 2018). However, colonies of social insects can differ not only in their average worker personality but also in worker personality distribution, which represents the level of among-individual variance within the colony. It was demonstrated that such variation has significant fitness consequences as, for example, higher intracolonial variance in aggression has a positive influence on ant colony productivity (Modlmeier and Foitzik 2011). Environmental variability can also induce changes in group behavior (Pinter-Wollman et al. 2012; Gordon et al. 2013; Bengston and Jandt 2014). Such plasticity may allow groups to cope with short-term environmental variation, but it is less well known if environmental conditions can create fixed or long-term effects on colony personality (Bengston and Jandt 2014). Although, the most probable scenario is a combination of all these factors (Pinter-Wollman 2012): mean colony personality, its distribution among colony members, and environmental influence.

Behavioral differences of group members can impact group performance and fitness (Wray et al. 2011; Bengston and Dornhaus 2014; Modlmeier et al. 2012, 2014; Blight et al. 2016). Moreover, individual behavioral traits, such as aggression, exploration, and boldness were found to have important fitness consequences in many different species (Smith and Blumstein 2008), including ants (Modlmeier and Foitzik 2011; Modlmeier et al. 2012). These studies also suggested that environmental factors such as habitat quality or population density can be associated with behavioral variation. Some papers have already shown the effect of climatic gradients on ant behavioral syndrome and its fitness consequences (Bengston and Dornhaus 2014; Segev et al. 2017). Contrary to previous studies, we wanted to directly analyze the relationship between habitats, behavioral traits, and productivity of ant colonies on various levels of social organization. We would like to emphasize that our study is the first one performed on social insects to demonstrate the effect of habitat type and other habitat-related factors on among- and intraindividual behavioral variation (i.e., behavioral plasticity, rIIV), but also within and among colonies in nest displacement efficiency.

For our study, we chose a metapopulation system, formed by ant colonies living in seminatural wet meadows and meadows invaded by *Solidago* sp. plants. Our previous studies performed on ants inhabiting these two habitat types demonstrated different brood investments (Grześ et al. 2018) and that *Myrmica* ant colonies living in invaded meadows are smaller in size and also have lower nest densities (Lenda et al. 2013; Grześ et al. 2018; Trigos-Peral et al. 2018). Moreover, workers have to forage over longer distances probably because protein resources are the main limiting factor in invaded meadows (Lenda et al. 2013; Trigos-Peral et al. 2018). Such differences among ant colonies suggest that different habitat types can be characterized by various environmental and colony traits, as colonies have to face various selective pressures. Furthermore, as a response to various selective pressures, different behavioral traits may affect differently the colony productivity in these two habitat types.

In this study, we studied four behavioral traits measured at individual-level (aggression and foraging behavior characterized by exploration, meandering, and activity) and one at colony level (nest displacement) in a *Myrmica rubra* ant metapopulation system inhabiting seminatural and invaded meadows. Our main goals were to test 1) the effects of various environmental and colony characteristics (habitat type and traits depending on the habitat type, as colony size, intra- and interspecific nest density, as well as the worker size) on the mean (i.e., among-individual and colony variation) and rIIV (i.e., intraindividual and colony rigidity) of behavioral traits measured at individual and colony levels; 2) whether there are consistent individual differences among habitats (i.e., behavioral types) and between-individual correlations among functionally different behavioral traits (i.e., behavioral syndromes). We also tested 3) whether the mean and variance of the behavioral traits measured at different levels (individual and colony) have an effect on the productivity parameters (different brood types) of ant colonies living in invaded and seminatural habitats.

Meadows invaded by *Solidago* plants seem to create more homogeneous environment thus we hypothesize lower behavioral trait variation of workers and colonies from this habitat type compared to ants living in seminatural meadows. According to the results of other studies (e.g., Modlmeier and Foitzik 2011; Maák et al. 2019), we also assumed that habitat and colony characteristics, mostly nest density and colony size will strongly influence both behavioral trait variation and plasticity of individuals and colonies. We also expected that the level of rIIV differs between habitats. As previous studies suggest, a positive interaction between individual state and rIIV exists (DiRienzo and Montiglio 2016; Lichtenstein et al. 2017), thus we expect larger individuals to express higher rIIV. Moreover, we hypothesized that habitat type will affect the behavioral types of workers and colonies and that functionally different behavioral traits will show different behavioral syndromes on individual and colony levels. Finally, we assumed that because of various selective pressures occurring in two habitat types, the effect of different behavioral traits on colony productivity will be different between the two habitat types. We assumed that in seminatural meadows, as a response to intraspecific competition, colonies with higher aggression level will have higher productivity, whereas in the invaded meadows, higher exploration skills and activity of workers will enhance the productivity of *Myrmica* colonies.

## MATERIAL AND METHODS

### Field data collection

The study was conducted at the beginning of August 2016 on the *M. rubra* metapopulation system occurring in grasslands near the city of Kraków (50°01′N/19°53′E) in a meadow complex occupying the flat-bed of the Vistula River valley, at an altitude of 200–240 m above sea level. Recently, many meadows have been invaded by goldenrod (*Solidago* spp.). Three seminatural meadows and three meadows invaded by goldenrod were randomly selected on the study site with a minimum distance of 1 km and a maximum distance of 5 km among meadows. Meadows were separated from each other by a watercourse, forest, and a human settlement. Seminatural meadows were those that had a surface covered 100% by native plants, mainly *Molinion caeruleae*, but also with a high abundance of *Sanguisorba officinalis* and some rare plant species, such as *Gentiana pneumonanthe*, *Gladiolus imbricatus*, *Iris sibirica*, or *Trollius europaeus*. Meadows invaded by goldenrods were nearly pure stands (covered 90–100%) of *Solidago* plants, with only a few other plant species (Moroń et al. 2009).

Twelve *M. rubra* colonies were collected from three meadows with the main plant community formed by the *Molinietum caeruleae* association (further on seminatural habitats) and 11 colonies were collected from three grasslands invaded by goldenrod (further on invaded habitats). Nests were separated by a distance of at least 20–30 m to cover a large area from each meadow in order to include potential habitat heterogeneity and to avoid the overlapping of the home ranges of focal colonies. We assessed the density of all other ant species nests around each *M. rubra* colony in a square of 9 m^2^, with the chosen *M. rubra* nest in the middle of the square. During the statistical analyses, we considered separately the number of *Myrmica* nests (med: 1, min/max: 0/9) and all the nests belonging to other species found around our focal colonies (further on the number of allospecific nests; med: 1, min/max: 0/3). About 10 ants from each of the found nests were taken to the laboratory for identification using the keys of Czechowski et al. (2012). Afterwards, all focal colonies were excavated and transferred to the laboratory. In all the cases, we excavated a larger area around the nests and ensured that no more ants were present in the surrounding area (such procedure was used in all nest dimensions).

### *Myrmica* ant colony size and productivity parameters

In the laboratory, for each colony we counted: 1) the number of adult workers (further on colony size; median: 1636, min/max: 219/5964) and 2) queens, 3) the number of ant larvae, 4) the number of ant pupae, which were divided into worker, male and queen (gyne) pupae, and 5) the number of winged queens and 6) males. The number of queens, larvae, and workers were correlated (Spearman rank correlation: 606.65 < S < 1117.9, *P <* 0.03, ρ > 0.45), so we decided to use only the number of workers (environmental factor at the individual-level but intrinsic at the colony level) in our statistical analysis. Information on the total number of larvae, pupae and winged sexual forms produced by the colony allowed us to calculate total colony production.

### Colony rearing

After assessing colony size, each *M. rubra* colony was placed and maintained in a plastic container (24 × 15 × 12 cm) under identical laboratory conditions for 3 weeks before the beginning of the behavioral assays. Box walls were coated with paraffin to prevent ants from escaping, whereas the bottom of the nest was filled with plaster. A small piece of wet sponge was added to each colony to maintain appropriate humidity, and it was covered by a flowerpot saucer with a notched entrance to provide a suitable and dark place for ants and their brood. Ants were fed twice per week with a 50% glucose solution and frozen fly larvae. All colonies were reared in their original size; therefore, the colonies were provisioned with specific amount of food according to their colony size.

### Behavioral observations

After three weeks of acclimatization, from each colony, we choose 12 older workers (foragers) on the basis of the melanization level of the cuticle (Cammaerts-Tricot 1974). Selected ants had a dark-brown colored cuticle on their head and gaster. According to the classification presented by Cammaerts-Tricot (1974), these workers were 4–6 months old. Workers were used in three behavioral assays: aggression, exploration, and locomotion. Each ant was individually marked using a personalized color combination on the thorax and abdomen with the help of Art Deco enamel paint markers. For the period of the behavioral assays, the selected ants were separated from the original colony and kept inside small plastic containers (18 × 12 × 6 cm) with a wet sponge covered by a flowerpot saucer. The behavioral tests were performed 24 h after the removal of the workers and were conducted over the three following days. Workers from the same colony were always tested for all behavioral assays on the same day with a random order of the behavioral tests. There were 1 h breaks between the assays performed on the same day. Altogether, we conducted three trial series, thus each worker was tested nine times (three times for aggression, three for exploration, and three for locomotion). In the analyses testing the effects of different characteristics on the mean- and residual variance of the behavioral traits, in those investigating the behavioral syndrome and consistency (repeatability), but also in models testing the effect of behavioral traits on the productivity parameters at individual-level, we included only workers that were tested nine times (three times per assay, *N*_*trials*_ = 681, *N*_*individuals*_ = 227: *N*_*seminatural*_ = 122, *N*_*invaded*_ = 105). In the analyses testing the effect of mean and SD of behavioral traits on productivity, we included all the workers and trials that were performed (*N*_*trials*_ = 750; *N*_*seminatural*_ = 400, *N*_*invaded*_ = 350). The reduced sample size was due to the death or escape of some of the individuals (*N*_*seminatural*_ = 22, *N*_*invaded*_ = 27) during behavioral observations. After the behavioral tests, we measured the head width of workers in order to express their body size (µm; further on worker size). The head of each ant was measured using a metallographic microscope under 100× magnification based on digital photographs taken with a digital camera (Panasis, ver. 2.4.2, Huvitz).

### Aggression assays

Aggression was measured by confronting each worker with a freshly defrosted dead non-nestmate worker coming from one of the other colonies used during the experiments. We used dead workers as opponents to eliminate behavioral differences between our stimuli and to concentrate our interest on the response of the worker. Ants were killed by freezing at −20 °C and were thawed 10 min before the experiments. We used a new corpse for every worker tested. Before each aggression assay, a plastic cylinder coated with fluon (diameter: 3 cm) was put inside the box to segregate a small space with the focal ant inside (after each test the cylinder was slightly replaced within the box to avoid the potential effects of chemical traces left behind by alive or dead ants). After 30 s, we gently introduced the defrosted corpse of a non-nestmate. The initial encounter, which was defined as the first behavior of the focal ant with the corpse, was recorded and scored as follows: fleeing (rapid movement of the focal ant in the opposite direction) = 0; antennation = 1; mandible opening = 2; biting or stinging = 3.

### Exploration assays

To measure how ants explore a new environment, we tested individual exploration ability (further on exploration). We used a transparent plastic box (18 × 12 × 6 cm) with a sheet of gridded paper (twenty-four 9 cm^2^ squares) fixed under its bottom. Before the start of each observation, the focal ant was carefully placed inside the tube (5.7 cm long Falcon plastic tube covered with aluminum foil), which was always placed at the same specific location (on the middle grid line in the left side of the box). The entrance of the tube was locked with a plastic cork for 2 min. After this time, the plastic cork was carefully removed, and we measured the time spent by the worker to emerge from the refuge. If the ant left the refuge, the number of new squares (exploration) that were entered during its path was recorded and used in future analysis. Each observation lasted for 3 min. The plastic boxes and tubes were cleaned with alcohol and changed between the trials. They were reused only after every fourth trial.

### Locomotion assays

Locomotory assays were performed to test the physical properties of ant movement. The locomotion of workers was studied by tracking the movement of a worker in a Petri dish (10 cm diameter). Before the video tracking, the ant was gently placed with the use of a soft pincette into a small plastic cylinder coated with fluon (diameter: 3 cm) in the center of the Petri dish. The ants were allowed to acclimatize for 2 min; after this period, the cylinder was removed and the individuals could freely move inside the dish. The movement of the ant was recorded for 3 min by using a Microsoft LifeCam Studio camera (1280 × 720 pixels resolution) placed 20 cm above the Petri dish. The locomotive behavior of each ant was analyzed by automated tracking software (EthoVision^®^ XT v. 12; Noldus Information Technology 2016). A threshold movement of 0.05 cm was used as an input filter to eliminate system noise or slight body movements that were not associated with locomotion (Bernadou et al. 2015). Two behavioral parameters were calculated from each digitized paths: 1) total distance traveled by an individual (further on activity) during the three minutes of observations (cm) and (2) meandering (°/cm; further on meandering): mean absolute change in the direction of movement of the ant relative to the distance moved (Bernadou et al. 2015). The Petri dishes were cleaned with alcohol and changed between the trials.

### Nest displacement assays

To assess nest displacement ability, we selected 15 old workers, 5 young workers (to ensure brood care) as well as 10 ant larvae of similar size from each colony and transferred them to a plastic box (30 × 16 × 10 cm). Inside the box, we prepared a flowerpot saucer nest as described before. After the ants were transferred, we waited 24 h for their acclimatization. Before the start of the experiment, we placed the same flowerpot saucer construction at the opposite end of the plastic box, which was followed by the removal of the old flowerpot saucer nest (to imitate nest destruction). The ants were removed by slight shakes and knocks, but without touching them with foreign objects. The observation started at the time of the removal of the old flowerpot saucer and we recorded the transport time of the first and last larvae into the new nest chamber. However, we used only the time of transport of the last larvae in further analysis (further on nest displacement efficiency) as it includes both important characteristics defining nest displacement efficiency (the time needed for new nest site discovery and the time span between the first and last larval transport) (see also Maák et al. 2019). We repeated this experimental procedure three times for each colony by selecting different workers and larvae each time and by using different boxes and nest chamber elements.

### Ethical note

The stress caused to ants during their collection in the field and their transport to the laboratory was minimized as much as possible. In the laboratory, colonies were maintained under nearly natural living conditions, thereby maximizing their welfare and survival. During the experiments, we performed only behavioral observations and non-invasive contacts with the ants. No individuals were intentionally harmed or subjected to stressful situations. After the end of the experiments, colonies were kept in the laboratory until their natural death.

### Statistical analyses

#### Effect of habitat and colony characteristics on mean- and residual variance

In order to model individual differences in among-individual variance and rIIV, we applied a double hierarchical general linear modeling (DHGLM) approach (see Westneat et al. 2013; Cleasby et al. 2015; Houslay and Wilson 2017). This method allows for iterations between two linear mixed-effect models, one explaining the mean and the other explaining residual dispersion, thus made possible the simultaneous modeling of the mean and residual variance level effects. We have to note here that based on three behavioral measurements per individual predictability estimates presented here might be of low precision (see Cleasby et al. 2015) and thus, our results regarding this analysis must be treated with caution. However, we believe our results are still informative in that individuals consistently differ in residual intraindividual variance and covariation between among- and intraindividual behavioral variance may arise. As this notion might be novel in the field of social-insect research, we find it useful to present this aspect of the study.

We fitted a mean model (equation 1.1) with the fixed effects of worker size (β _1_), colony size (β _2_), number of *Myrmica* nests (β _3_), number of allospecific nests (β _4_), habitat type (β _5_; factor with levels 0 or 1), and number of repeats (number of behavioral assays; β _6_). The model also included the random intercept effect of individual identity (ID), giving a predicted value for the intercept of each ID_j_ (j = 1: N_ID_). We also fitted a random intercept of colony identity to control for colony effects, giving a predicted value for each of the 23 colonies (k) as a deviation from the fixed effects. We defined alternative models in the *lme4* R package (Bates et al. 2015) and examined their goodness of fit relative to the model described in equation 1.1 by using likelihood ratio test. These investigations revealed that both random intercept terms are significant (*P* < 0.001 for both *ID*_*µj*_ and colony *k*).

The residual model (equation 1.2) was fitted with the fixed effects of worker size, colony size, number of *Myrmica* nests, number of allospecific nests and habitat (with “γ” representing fixed effect coefficients), and also a random intercept of ID, that modeled individual differences in rIIV. Following the methods of Mitchell et al. (2016), we allowed for a covariance (equation 1.3) between predicted mean values of activity (*ID*_*µj*_, equation 1.1) and predicted log-standard deviation ($ID_{\sigma_{\varepsilon }}$_*j*_, Equation 1.2) among individuals.

$$\begin{array}{l} \begin{array}{ll} & \mu_{jk}∼\beta_{0}+\beta_{1}\;worker\;size\;+\beta_{2}\;colony\;size\;\\ & +\beta_{3}Myrmica\;nests\;+\beta 4\;allospecif\;ic\;nests\;\\ & +\beta_{5}\;habitat\;type\;+\beta_{6}\;repeats\;+ID_{\mu j}+\;colony_{k} \end{array} \end{array}$$ (1.1)

$$\begin{array}{l} \begin{array}{ll} & log_{e}{\left(\sigma_{\varepsilon }\right)}_{j}∼\;\gamma_{0}+\;\gamma_{1}\;worker\;size\;+\gamma_{2}\;colony\;size\;\\ & +\gamma_{3}\;Myrmica\;nests\;+\gamma_{4}\;allospecif\;ic\;nests\;\\ & +\gamma_{5}\;habitat\;type\;{+{ID}_{\sigma \varepsilon j}}_{,} \end{array} \end{array}$$ (1.2)

$$\begin{array}{l} ID_{\sigma \varepsilon j}∼\;MVN\left(0,\;\Omega \right):\;\Omega_{ID}=\;\left[\begin{array}{ll} \sigma_{ID_{\mu }}^{2} & \\ COV_{ID_{\mu },ID_{\sigma }} & \omega_{ID_{\sigma_{\varepsilon }}}^{2} \end{array}\right] \end{array}$$ (1.3)

We analyzed each behavioral trait separately. For nest displacement, we could estimate variance on the between-colony and within-colony level, thus the models were built in the same way as described above, the only difference being that colony identity was fitted as a sole random intercept. Aggression, meandering, and nest displacement were log-transformed to achieve normality. Behavioral scores and continuous fixed effects were centered (standardized to mean = 0, standard deviation = 1) to aid model fitting and to facilitate comparison of variance parameters. Therefore, variances of random intercepts in the mean model can be interpreted as proportions of the total phenotypic variances of the dataset. The normality of random effects and the residual variation were checked visually in plots of predicted random effect values fitted versus residual values. All parameters were given uninformative priors. Model code is available via the Supplementary Material.

### Behavioral syndrome and consistency

To test for among-trait (co)variation at the among-colony, among-individual, and intraindividual (residual) level, we ran two separate multivariate mixed-effect models. In the first model, the four individual-level behavioral traits were fitted as response variables, habitat type as an explanatory factor, while “colony” and “individual” were random effects. A second model was fitted to estimate among-colony and within-colony (co)variation across nest displacement and the other behavioral traits. However, from this model, only the among-colony (co)variation of the behavioral traits and the nest displacement was taken into account, as the within-colony (residual) (co)variation was measured on different levels in the traits. In this model, the five behavioral traits were response variables, habitat type as an explanatory factor, and “colony” was used as a random effect. Based on our model, we decomposed phenotypic correlations into among-colony, among-individual, and intraindividual (residual) correlations, using among-colony and among-individual phenotypic correlations as an indicator of behavioral syndromes (see Garamszegi et al. 2012; Herczeg and Garamszegi 2012; Dingemanse and Dochtermann 2013). The results are given as correlation coefficients and their 95% credibility intervals.

The consistency of each behavioral trait at the colony (12 individuals per colony) and individual (3 repetitions per individual) level were calculated with intraclass correlations (R_ICC_, Lessels and Boag 1987) by using LMM-based (Gaussian data fit, *N*_*bootstrap*_*=* 1000) calculations (Nakagawa and Schielzeth 2010) with colony and individual identity as random factors.

### Colony and individual behavioral traits affecting productivity parameters

The effects of themean and the SD (referring to the among-individual variance) of colony level behavioral traits on the total production of the colonies were analyzed only altogether for the two habitats. In the GLM (negative binomial error, maximum likelihood fit, *N* = 23), total production was included as dependent variable, habitat type was included as a fixed factor, while the mean (aggression, exploration, activity, meandering, and nest displacement) and SD (aggression, exploration, activity, meandering, and nest displacement) of the behavioral traits were included as covariates/explanatory variables. The same model construction but separate models were used for testing the effect of different behavioral traits on the number of new gynes (gyne pupae + winged), males (gyne pupae + winged), and workers (larvae + pupae). Considering the period of the year and species biology (Radchenko and Elmes 2010), all the larvae were treated as worker larvae.

The effects of individual behavioral traits (measured at the individual-level) on total colony production were analyzed with GLMMs (negative binomial error, maximum likelihood fit, *N*_*total*_*=* 681), except the total production in invaded meadows, where LMM was used (Gaussian error, maximum likelihood fit; *N* = 681). In the full models, behavioral traits were included as fixed effects, while individual ID as a combined random factor. The same model construction but separate models were used for testing the effect of different behavioral traits on the number of new gynes (gyne pupae + winged), males (male pupae + winged), and workers (larvae + worker pupae). Having information already about the effect of different habitat type (invaded vs. seminatural) on colony productivity parameters (Grześ et al. 2018), the analyses on the effect of the different individual behavioral traits were conducted separately for the two habitats types (*N*_*seminatural*_ = 366, *N*_*invaded*_ = 315). New males were found only in a low number of colonies from the invaded habitats, so the results of these analyses were not included.

All statistical analyses were carried out in the R Statistical Environment (R Core Team 2019). Models were fitted in the Bayesian, Markov Chain Monte Carlo software JAGS (Plummer 2003), through the *rjags* interface (Plummer et al. 2019). Multivariate mixed models were fitted using the *MCMCglmm* function from the *MCMCglmm* R package (Hadfield 2010), which implements a Bayesian framework for model fitting with long iterations (1 300.000 with 300 000 burn-in periods); the Markov chain was sampled at each 1000th iteration. Consistency was calculated with the *rpt* function (*rptR* package, Stoffel et al. 2017). All LMMs and GLMMs were performed using the *lmer* or *glmer* function, respectively (*lme4* package, Bates et al. 2013), automated model selection with the help of the *dredge* function (*MuMIn* package, Bartoń 2013). If models were overdispersed, negative binomial error structure was applied (see Lindén and Mäntyniemi 2011). Linear models were performed using the *lm* function; GLMs with the *glm.nb* function and automated model selection with the help of the *stepAIC* function (*MASS* package, Venables and Ripley 2002). All variables were standardized before the analysis (Gotelli and Ellison 2004).

## RESULTS

### Effect of habitat and colony characteristics on mean- and residual variance of ant behavior

Based on our models, mean individual activity (described by total distance traveled) became lower across the trials (Table 1). Both random effect terms (individual and colony ID) explained variation in the mean activity (activity had an individual and a colony-specific expression; Table 1). We found that individual activity was less predictable (i.e., high rIIV) when more *Myrmica* nests were around (Table 1), while the pattern was the opposite regarding the number of allospecific nests around the focal colonies (Table 1). Individual random effects term explained variation (substantial among-individual variation) in rIIV (Table 1). For the remaining nonsignificant effects, see Table 1. We found no significant correlation between individual mean and rIIV of activity (r_Int,rIIV_ = 0.21 [−0.13–0.56]).

**Table 1:** Sources of variation in activity, exploration, meanderness, and aggression of *M. rubra* ants. Estimates were derived from a double hierarchical general linear model

	Activity	Meanderness	Exploration	Aggression
Model	Posterior mean (95% CrI)	Posterior mean (95% CrI)	Posterior mean (95% CrI)	Posterior mean (95% CrI)
(a) Mean	β	β	β	β
Intercept	−0.11 (−0.75–0.58)	0.13 (−0.59–0.85)	−0.1 (−0.81–0.69)	0.35 (−0.23–0.51)
Worker size	−0.06 (−0.18–0.06)	0.007 (−0.07–0.09)	0.05 (−0.06–0.15)	−0.03 (−0.15–0.02)
Colony size	0.16 (−0.07–0.38)	−0.072 (−0.31–0.14)	0.14 (−0.31–0.37)	**0.11 (0.007–0.21)**
Nr. of *Myrmica* nests	0.04 (−0.21–0.28)	−0.067 (−0.29–0.16)	0.04 (−0.21–0.29)	−0.004 (−0.19–0.25)
Nr. of other nests	−0.22 (−0.45–0.007)	0.21 (−0.005–0.44)	−0.09 (−0.32–0.15)	−0.14 (−0.38–0.03)
Habitat type	0.08 (−0.38–0.49)	−0.15 (−0.61–0.32)	0.03 (−0.64–0.54)	−0.29 (−0.42–0.15)
Nr. of repeats	**−0.17 (−0.21** to **−0.12)**	0.01 (−0.02–0.04)	−0.01 (−0.05–0.04)	0.004 (−5.570e−06–0.07)
	σ ^2^	σ ^2^	σ ^2^	σ ^2^
Individual (random intercept)	**0.58 (0.45**–**0.67)**	**0.36 (0.28**–**0.44)**	**0.45 (0.38**–**0.54)**	**0.57 (0.48**–**0.65)**
Colony (random intercept)	**0.43 (0.27**–**0.66)**	**0.43 (0.22**–**0.66)**	**0.49 (0.24**–**0.86)**	**0.61 (0.36**–**0.9)**
(b) Residual variation	γ	γ	γ	γ
Intercept	**−0.69 (−0.98 to −0.37)**	**−1.02 (−1.44 to −0.6)**	**−0.44 (−0.75 to −0.15)**	**−7.82 (−11.06 to −0.18)**
Worker size	0.03 (−0.06–0.12)	−0.06 (−0.18–0.07)	−0.06 (−0.15–0.04)	0.19 (−0.82–1.21)
Colony size	−0.07 (−0.16–0.01)	−0.13 (−0.25–0.002)	**−0.16 (−0.26 to −0.07)**	−0.26 (−1.8–1.04)
Nr. of *Myrmica* nests	**0.097 (0.01**–**0.18)**	0.02 (−0.11–0.16)	−0.05 (−0.15–0.05)	−0.35 (−1.19–0.49)
Nr. of other nests	**−0.098 (−0.19 to −0.001)**	0.07 (−0.05–0.2)	0.01 (−0.08–0.1)	−0.04 (−0.36–0.46)
Habitat type	0.13 (−0.06–0.32)	0.21 (−0.07–0.48)	0.05 (−0.15–0.24)	−0.98 (−2.99–1.24)
	σ ^2^	σ ^2^	σ ^2^	σ ^2^
Individual (random intercept)	**0.26 (0.01**–**0.42)**	**0.7 (0.59**–**0.81)**	**0.4 (0.31**–**0.49)**	**7.68 (0.07**–**9.99)**

Worker size, colony size, number of nearby *Myrmica* nests, number of nearby allospecific nests, habitat type (factor with two levels: *Seminatural* vs. *Solidago*) and Day (day of behavioral trial) were fitted as fixed effects without interactions. Posterior means and 95% credible intervals (CrI) are shown. Effects strongly supported by the model (95% CI not overlapping) are in bold font. Effects on (a) means and (b) the residual variation.

Mean and rIIV of individual meanderness was not affected by any of the habitat and colony characteristics. Meanderness had an individual and a colony-specific expression (Table 1). There was substantial among-individual variation in rIIV (Table 1). For the remaining nonsignificant effects, see Table 1. More meandering individuals also tended to be less predictable (r_Int,rIIV_ = 0.98 [0.61–0.81]).

The mean individual exploration was not affected by any of the fixed effects (Table 1); however, it had both an individual and a colony-specific expression (Table 1). Individual exploration was more predictable (i.e., low rIIV) with larger colony size (Table 1). There was also a substantial among-individual variation in rIIV (Table 1). For the remaining nonsignificant effects, see Table 1. Individuals with larger rIIV (i.e., low predictability) tended to be less explorative (r_Int,rIIV_ = −0.96 [−0.99 to −0.89]).

**Figure 1:**
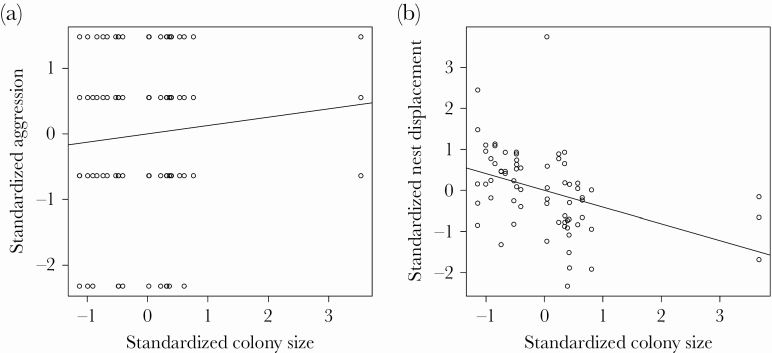
The effect of standardized colony size on the standardized aggression (A) and standardized nest displacement efficiency (B). The black lines are plotted using the formula of linear regression (y ~ x).

The individual mean aggression increased with colony size (Table 1; Figure 1A). Moreover, aggression had both an individual and a colony-specific expression (Table 1). The residual level of aggression was not affected significantly by any of the fixed effects, but there was a substantial among-individual variation in rIIV (Table 1). We found no significant correlation between individual mean behavior and rIIV of aggression (r_Int,rIIV_ = 0.09 [−0.17–0.38]).

The only behavior estimated on the colony level was nest displacement efficiency. According to our model, the mean nest displacement efficiency increased with colony size (Figure 1B) and with allospecific nest density (Table 2). Nest displacement had a colony-specific expression (Table 2). Moreover, there was also a substantial among-colony variation in the residual variation (among-individual variation in this case; Table 2). We found no significant correlation between among- and within-colony variation in nest displacement (*r* = −0.35 [−0.99–0.73]). For the remaining nonsignificant effects, see Table 2.

**Table 2:** Sources of variation in nest displacement behavior of *M. rubra* ants. Estimates were derived from a double hierarchical general linear model

Model	Posterior mean (95% CrI)
(a) Mean	β
Intercept	0.11 (−0.74–0.91)
Head size	−0.1 (−0.41–0.22)
Colony size	**−0.49 (−0.86 to −0.18)**
Nr. of *Myrmica* nests	0.06 (−0.27–0.4)
Nr. of allospecific nests	**−0.32 (−0.61 to −0.02)**
Habitat type	−0.09 (−0.59–0.43)
Nr. of repeats	−0.1 (−0.26–0.07)
	σ ^2^
Colony (random intercept)	**0.34 (0.08**–**0.67)**
(b) Residual variation	γ
Intercept	−0.03 (−1.17–0.97)
Head size	0.06 (−0.32–0.44)
Colony size	0.12 (−0.22–0.48)
Nr. of *Myrmica* nests	0.14 (−0.23–0.53)
Nr. of allospecific nests	−0.02 (−0.42–0.4)
Habitat type	−0.19 (−0.85–0.52)
	σ ^2^
Colony (random intercept)	**0.5 (0.16**–**0.95)**

Head size, colony size, number of nearby *Myrmica* nests, number of nearby allospecific nests, habitat type (factor with two levels: *Seminatural* vs. *Solidago*) and Day (day of behavioural trial) were fitted as fixed effects without interactions. Posterior means and 95% credible intervals (CrI) are shown. Effects strongly supported by the model (95% CI not overlapping) are in bold font. Effects on (a) means and (b) the residual variation.

### Behavioral syndrome

We found no effect of habitat type on the among-trait (co)variation measured at individual (effect = 0.07, CrI [−0.16–0.37], pMCMC = 0.52) and colony (effect = 0.02, CrI [−0.19–0.3], pMCMC = 0.94) levels. The behavioral syndrome at individual-level involved all the studied traits (activity, exploration, aggression, and meanderness; Table 3). Individuals that were more active, were also more explorative and aggressive, whereas showed a less meandering pathway. However, at colony-level, we found a behavioral syndrome related to foraging involving activity, exploration, and meanderness (Table 3). Within individuals, only the activity and meanderness were correlated (Table 3).

**Table 3:** Correlations among behavioral traits: *r* (95% CrI)

Behavioral traits	Level	*r* (95% CrI)
Activity – Exploration	**Between colonies**	**0.89 (0.77**–**0.97)**
	**Between individuals**	**0.91 (0.72**–**0.97)**
	Residual	0.02 (−0.08–0.08)
Activity – Aggression	Between colonies	0.15 (−0.17–0.56)
	**Between individuals**	**0.35 (0.19**–**0.51)**
	Residual	−0.04 (−0.18–0.02)
Activity – Meanderness	**Between colonies**	**−0.83 (−0.95** ** to ** **−0.65)**
	**Between individuals**	**−0.89 (−0.92 to −0.82)**
	**Residual**	**−0.6 (−0.68** ** to ** **−0.56)**
Activity – Nest displacement	Between colonies	−0.09 (−0.46–0.32)
Exploration – Aggression	Between colonies	0.28 (−0.23–0.54)
	**Between individuals**	**0.35 (0.17**–**0.49)**
	Residual	0.027 (−0.04–0.13)
Exploration – Meanderness	**Between colonies**	**−0.76 (−0.9** ** to ** **−0.58)**
	**Between individuals**	**−0.86 (−0.91 to −0.68)**
	Residual	−0.003 (−0.07–0.96)
Exploration – Nest displacement	Between colonies	−0.1 (−0.41–0.42)
Aggression – Meanderness	Between colonies	−0.23 (−0.59–0.3)
	**Between individuals**	**−0.27 (−0.49 to −0.19)**
	Residual	−0.029 (−0.12–0.06)
Aggression – Nest displacement	Between colonies	−0.01 (−0.33–0.32)
Meanderness – Nest displacement	Between colonies	0.24 (−0.39–0.47)

Effects strongly supported by the model (95% CI not overlapping) are in bold font.

### Behavioral consistency

The colony-level consistency of the studied behavioral traits was low but significant for all of the studied behavioral traits (Table 4). The lowest consistency values at the colony-level were found for aggression (*R* = 0.13), whereas the highest for nest displacement efficiency (*R* = 0.41; Table 4). On the other hand, the consistency of the behavioral traits was much higher at individual-level with the lowest value found for exploration (*R* = 0.22; Table 4).

**Table 4:** Repeatability estimates for the studied behavioral traits altogether for the two habitat types on colony- and on individual-level of *M. rubra* ants. Repeatability values (R) and 95% CI are shown. Significance (*P*) estimates are based on randomization tests

Behavioral traits	Colony-level *N* = 68; R (95% CrI)	Individual-level *N* = 681; R (95% CrI)
Activity	**0.2 (0.087**–**0.315)**	**0.33 (0.242**–**0.419)**
Aggression	**0.13 (0.044**–**0.223)**	**0.27 (0.181**–**0.352)**
Exploration	**0.18 (0.074**–**0.288)**	**0.22 (0.142**–**0.303)**
Meandering	**0.25 (0.117**–**0.378)**	**0.28 (0.203**–**0.371)**
Nest displacement	**0.41 (0.125**–**0.636)**	

Effects strongly supported by the model (95% CI not overlapping) are in bold font.

We also considered behavioral consistency separately for the two habitat types. In the seminatural habitat, colony-level consistency (*R* = 0.16–0.37) became higher than in the invaded meadows (*R* = 0.11–0.13; Supplementary Table S1), except nest displacement efficiency (*R*_seminatural_ = 0.36; *R*_invaded_ = 0.42; Supplementary Table S1). On the other hand, the individual-level consistency became lower (every behavioral trait: *R* = 0.16–0.28) compared to the invaded meadows (*R* = 0.27–0.38; Supplementary Table S1).

### Colony behavioral traits affecting productivity parameters

**Figure 2:**
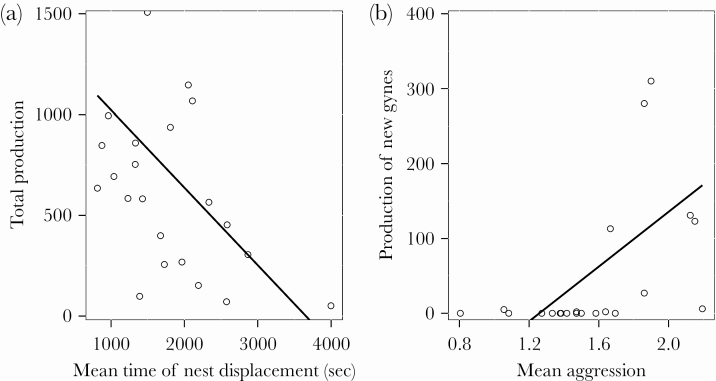
The effect of the mean nest displacement (A) and aggression (B) on the colony-level production parameters (total production: A, new gynes: B). The black lines are plotted using the formula of linear regression (y ~ x).

The total production of *Myrmica* colonies was higher with higher among-individual variation of workers (SD) in the terms of aggression, mean exploration, and mean meanderness but became lower with the higher mean (Figure 2A) and variability (SD) of nest displacement, and variability of the meanderness (Table 5). The production of workers was positively affected by the mean exploration and meanderness but also when colonies were more variable (SD) in terms of aggression and activity (Table 5). On the other hand, mean and higher variability of nest displacement but also higher variability of meanderness had a negative effect on the production of new workers (Table 5). The production of new gynes was affected positively by the mean for aggression (*z =* 3.2, *P* = 0.001; Figure 2B). Mean exploration (*z = -*1.05, *P* = 0.29) and mean activity (*z =* 1.24, *P* = 0.22) were also included in the best model, but their effect was not significant.

**Table 5:** Mean (colony level) behavioral traits of *M. rubra* workers affecting the total production and the production of new workers

Fixed effects	Total production effect (95% CrI)	Worker pupae effect (95% CrI)
Intercept	**6.39 (6.16**–**6.62)**	**6.31 (6.08**–**6.53)**
Habitat type	−0.28 (−0.64–0.07)	**−0.38 (−0.73 to −0.03)**
Mean activity	−0.23 (−0.61–0.14)	−0.3 (−0.67–0.07)
SD activity	0.24 (−0.03–0.52)	**0.29 (0.02**–**0.56)**
Mean meanderness	**0.88 (0.07**–**1.68)**	**0.93 (0.13**–**1.72)**
SD meanderness	**−0.95 (−1.68 to −0.22)**	**−1.01 (−1.73 to −0.3)**
Mean exploration	**0.73 (0.27**–**1.18)**	**0.82 (0.37**–**1.27)**
SD exploration	−0.2 (−0.42–0.02)	−0.18 (−0.4–0.03)
Mean aggression	−0.13 (−0.42–0.16)	−0.23 (−0.52–0.06)
SD aggression	**0.25 (0.06**–**0.44)**	**0.24 (0.05**–**0.42)**
Mean nest displacement	**−0.29 (−0.56 to −0.02)**	**−0.29 (−0.56 to −0.03)**
SD nest displacement	**−0.44 (−0.76 to −0.11)**	**−0.48 (−0.8 to −0.16)**

Effects strongly supported by the model (95% CI not overlapping) are in bold font.

### Individual behavioral traits affecting the production parameters

In the seminatural habitats, total production and the production of new workers were positively affected by workers’ aggression and exploration, and negatively by meanderness (Table 6). The production of new gynes was also positively affected by workers’ aggression and negatively by the meanderness (Table 6). In the invaded habitat, total production and the production of new workers were positively affected by workers’ activity and exploration, while total production was affected negatively only by meandering (Table 6). The production of new queens was not affected by any of the behavioral traits (Table 6).

**Table 6:** Individual behavioral traits of *M. rubra* workers affecting the production parameters

Behavioral traits	Total production effect (95% CrI)	New queens effect (95% CrI)	New workers effect (95% CrI)
Seminatural meadows			
Intercept	**6.45 (6.37**–**6.53)**	**2.5 (1.77**–**3.23)**	**6.34 (6.26**–**6.42)**
Activity	−0.003 (−0.1–0.09)	0.37 (−0.16–0.89)	−0.02 (−0.21–0.03)
Meanderness	**−0.12 (−0.21 to −0.03)**	**−4.67 (−8.04 to −1.3)**	**−0.12 (−0.21**–**0.03)**
Exploration	**0.13 (0.04**–**0.22)**	0.32 (−0.13–0.77)	**0.13 (0.04**–**0.22)**
Aggression	**0.17 (0.08**–**0.26)**	**0.38 (0.008**–**0.75)**	**0.15 (0.07**–**0.23)**
Invaded meadows			
Intercept	**6.37 (6.29–6.45)**	**4.25 (2.22**–**6.28)**	**6.52 (6.44**–**6.6)**
Activity	**0.18 (0.08**–**0.28)**	0.2 (−2.5–2.9)	**0.25 (0.15**–**0.34)**
Meanderness	−0.07 (−0.16–0.022)	*−*0.29 (−1.91–1.34)	−0.08 (−0.18–0.02)
Exploration	**0.14 (0.05**–**0.23)**	0.07 (−2.32–2.46)	**0.17 (0.06**–**0.26)**
Aggression	0.009 (−0.08–0.095)	0.2 (−2.07–2.47)	−0.04 (−0.13–0.05)

Effects strongly supported by the model (95% CI not overlapping) are in bold font.

## DISCUSSION

Our study shows, for the first time in social insects, the effects of environmental and colony characteristics on the mean- and residual variance of ant behavior measured at the individual and colony levels. All the studied behavioral traits showed substantial among-individual and colony variation, indicating repeatable differences in these levels in *M. rubra* ants. Workers and colonies also exhibited within-individual/colony variation linked to external variables and so showed phenotypic plasticity. Moreover, workers and colonies differed also in their residual variation. Although habitat type did not have a direct effect on the means or residual variation of studied behavioral traits, the different, habitat-dependent environmental and colony characteristics affected both the between and within individual/colony behavioral variation.

One of the most important factors influencing ant behavior seems to be colony size. We found that in larger colonies the mean forager aggression is higher compared to smaller colonies and also the mean nest displacement efficiency increased with colony size. Moreover, exploration was more predictable within individuals of larger colonies. Nest density is also affecting mean and variation of *Myrmica* ant behavior to a high degree. The nest displacement efficiency increased and the individual activity was more predictable when more allospecific nests were around focal colonies, however, the higher number of *Myrmica* nests resulted in less predictable individual activity. More predictable individuals were also less meandering and more explorative. Our results clearly demonstrated the existence of behavioral variation among *Myrmica* workers (foragers) in activity, meanderness, exploration, and aggression as well as variation among *Myrmica* colonies for the nest displacement ability. However, the individual and colony personality did not differ between the two studied habitats, suggesting low plasticity in the behavior. The behavioral traits show a habitat type-dependent role in determining colony productivity.

Invasive plants can induce negative changes in ground-dwelling arthropod assemblages (Schirmel et al. 2011; Gallé et al. 2015), usually by altering habitat structure (Hejda et al. 2009), but also by changing the nesting site (Somogyi et al. 2017) or food availability (Lenda et al. 2013; Trigos-Peral et al. 2018). The results of our previous studies performed on *M. rubra* ants living in meadows invaded by *Solidago* plants (Lenda et al. 2013; Grześ et al. 2018; Trigos-Peral et al. 2018) suggest that there can be different selective pressures on ant colonies living in these two habitat types thus we expected to find the differences in individual and/or colony personalities. Our results did not demonstrate such differences but found that the behavior of *Myrmica* workers and colonies can differ at three levels of variation (among- and intraindividual variation, and also in residual variation). This suggests that individual ants and colonies follow various behavioral strategies, which can be influenced by several environmental and colony characteristics. Heterogeneous residual variance of the activity and exploration shows heterogeneity in stochasticity (predictability) caused by colony size and nest density. Higher *Myrmica* nest density results in lower predictability of individual activity but allospecific nest density had an opposite effect (foragers had higher predictability in their activity). This can highlight the importance of competition for food and nesting sites which has an enhanced effect on seminatural meadows (see also Lenda et al. 2013; Grześ et al. 2018). Competition is usually considered to have a significant effect in shaping ant communities (Savolainen and Vepsäläinen 1988; Braschler and Baur 2003; Trigos-Peral et al. 2016), and unavoidably occurs when the ecological requirements of species overlap (Pianka 1974; Glen and Dickman 2008). The negative effects of competitive interactions can be reduced if the morphological, behavioral, and ecological plasticity of the competing partners allows shifts in their requirements, thus minimizing niche overlap, as observed also in ants (see Cerdá et al. 2013 for a review). The overlap of the requirements is stronger in colonies of the same species (Cerdá et al. 2013) that can lead to a higher variability in the activity of foragers. Higher individual variation in the activity can be beneficial for foraging success in the places with higher intraspecific competition and may be related to the collective regulation of the foraging response driven by environmental feedback (Gordon et al. 2011).

On the other hand, the neighboring ant colonies belonging to alien species have the opposite effect, and the activity of foragers becomes less variable as they can follow more similar strategies. This can be because *Myrmica* species are morphologically and behaviorally different from the *Formicinae* species and can have also different foraging strategy and activity period (Savolainen and Vepsäläinen 1988; Czechowski et al. 2012). The higher number of allospecific nests led also to an increased mean colony nest displacement efficiency. This behavior was also influenced by the colony size, as nest displacement efficiency was higher in larger colonies (considering the transport of the same amount of larvae). This result confirms our previous findings on *Myrmica scabrinodis* ants (Maák et al. 2019) that larger colonies can be more efficient due to the speed of discovering new nest sites, which can be linked to the higher number of scouts (Dornhaus and Franks 2006; Maák et al. 2019). Usually, more explorative foragers can acquire important information about the state of the colony and its environment, for example, the location of food sources (Herbers and Choiniere 1996), also enhancing in this way nest movement efficiency. In our study, colony size affected individual variation on exploration and nest displacement efficiency.

Body size may be the most important factor explaining variation in behavior, life history, and ecology of an organism; therefore, colony size can be an important determinant of collective behavior, colony development, and other traits (see Dornhaus et al. 2012 for a review, Juhász et al. 2020). Larger colony size can cause the evolution of distinct behavior, morphology, or other traits of individuals as a result of differing constraints and selection pressures (Dornhaus et al. 2012). Underpinning this, contrary to the size of the workers that did not have any significant effect on the behavioral trait variation, the colony size had a positive influence on the mean individual aggression and resulted in a lower variability (higher predictability) of exploration. Contrary to our results, no significant effect of the colony size on any studied behavioral traits was found in different *Temnothorax* species (Modlmeier et al. 2012; Bengston and Dornhaus 2014; Segev et al. 2017), except for the exploration of the environment (Modlmeier et al. 2012). In *M. scabrinodis*, we also did not find any effect of the colony size on the aggression of workers, although subcolonies with higher young-worker ratio showed higher aggression (Maák et al. 2019). This finding suggests that the defense of the colony can be regarded as a decision-making process based on a quorum, where all the participants perceive the ratio of experienced nestmates and adjust their own aggression accordingly (e.g., Hölldobler and Wilson 1990; Gordon 2010). It seems that a similar situation can occur also in larger colonies, where old foragers respond to the decrease of their ratio with higher aggression.

Larger colonies also showed a lower residual variability in exploration. Besides being more explorative, more predictable individuals were also less meandering, suggesting a straighter path for individuals with lower variance in exploration. Exploration is the strongest predictor of colony foraging success and can show consistent between-colony differences (Pasquier and Grüter 2016). Having in mind that larger colonies can have a higher food demand (e.g., Hölldobler and Wilson 1990; Dornhaus et al. 2012), this can lead to higher individual efficiency and sturdiness of workers (Dornhaus et al. 2012). This is underpinned by some findings in red-winged blackbirds (*Agelaius phoeniceus*), where it was found that the residual variability in the amount of food delivered decreased with food demand (older nestlings) and with trips out of the territory (on novel sites situated further) (Westneat et al. 2013). Our results underline the importance of taking into account not only the dissimilarities of biology and the habitat requirements of different species, but also of different populations (Gordon 2014, 2016), that can result in different levels of phenotypic variance (Westneat et al. 2015).

### Behavioral syndrome and consistency

All the studied behavioral traits had an individual and/or a colony-specific expression and showed a substantial among-individual/-colony variation in rIIV. This is strengthened further by our findings related to the personality. The personality of individuals involved all the behavioral traits (activity, exploration, aggression, and meanderness), whereas the colony personality involved activity, exploration, and meanderness. Such a complex behavioral syndrome was also found for *Myrmica ruginodis* ants at different levels of organization (Chapman et al. 2011), showing that patrolling individuals were significantly more active, bolder, and more aggressive than brood carers and foragers. At caste level, a boldness–aggression syndrome was described in patrollers, whereas a sociability–boldness syndrome was found at colony-level (Chapman et al. 2011). Moreover, colonies also showed strong internal concordance in the mean behavior (Chapman et al. 2011). For *M. rubra*, it seems that more mobile foragers are also more explorative and aggressive, but their pathway is less meandering. Chapman et al. (2011) found differences in the behavioral syndrome of *Myrmica* ants at individual-level in terms of the patroller caste, however, as with our results, they did not find personality levels in foragers. Our results suggest that these workers fit better with the more general syndrome situated on the “proactive–reactive axis” that has been found in a number of species (Sih, Bell, Johnson 2004; Blight et al. 2016), and is also present at different levels of the colony organization of social insects. Some individuals might be very aggressive and exploratory (proactive), while others could be more shy and cautious (reactive). Similar to solitary animals, proactive colonies are expected to be very active and flourish in stable environments, while reactive ones are better in adapting to changes in the environment (Sih, Bell, Johnson, et al. 2004; Blight et al. 2016). However, we did not find any difference in behavioral syndrome between ants from the two studied habitats that might be related to the characteristics of the behavioral traits that we chose for our study. This behavioral syndrome is showing lower plasticity probably because of its higher importance for individuals of the forager caste in every habitat, as their role is to explore the surrounding landscape, search for food items, and defend the colonies against predators or non-nestmates (see Hölldobler and Wilson 1990).

This behavioral syndrome can have also important fitness consequences (Biro and Stamps 2008), as it is also present at the colony-level of *M. rubra ants* (except aggression). Similarly, a behavioral syndrome was also found in other ant species, where colonies composed of more aggressive individuals were found to forage more effectively (Blight et al. 2016; Lichtenstein et al. 2016), explore their environment more thoroughly, and they were also more risk-prone, bold, and better intraspecific competitors compared to more docile colonies (Blight et al. 2016). Bengston and Dornhaus (2014) found consistent differences among colonies in coping style (involving foraging effort, foraging distances, and aggression), as some were more risk-prone, whereas others were more risk-aversive. The variation in group behaviors can be a product of both the environment and genetic factors and deciphering the relative contribution of these on collective behaviors is central in understanding its evolution (Wright et al. 2019). It seems that the traits of our analysis show a trade-off between these two effects (environmental and genetic).

In general, behavioral traits were slightly more consistent at the individual-level than at the colony-level (except nest displacement efficiency), but as these traits were measured at individual-level, this is not surprising. Despite this, we found a quite even consistency for every behavioral trait, except in aggression at colony-level. An opposite trend was found for the consistency of the behavioral traits at colony-level in *Temnothorax* ants, where exploration had low consistency, whereas aggression had a high level of consistency (Modlmeier et al. 2012). This was explained by a higher environmental influence on exploration and the strong genetic influence on aggression (Modlmeier et al. 2012). In foraging, which also includes exploration, the early experience of a worker can be highly influential (Ravary et al. 2007), but age polyethism (Seeley 1982) and age-related experience can also be important in this respect (Herbers and Choiniere 1996; Jeanson and Weidenmüller 2014; Gordon 2016). In our study, it seems that *M. rubra* colonies, but also individuals, may be under strong selective pressure for skills related to foraging (activity, exploration, and meanderness), as there is strong competition among ant colonies in seminatural meadows, whereas in invaded meadows, there is food scarcity and the colonies tend to be located close to a food source in an attempt to reduce foraging distances due to the unsuitability of the habitat (Trigos-Peral et al. 2018). Therefore, well-developed exploratory skills can increase colony fitness in both habitat types. On the other hand, the different selective pressures on ant colonies living in these two habitat types led to various trends in behavioral consistency which was higher on colony-level in seminatural meadows compared to invaded meadows but opposite tendencies were found for the individual-level. The higher colony-level variability in the invaded habitats may be linked to the more opportunistic occurrence of food sources and nesting sites but also other biotic and abiotic factors (see Lenda et al. 2013; Trigos-Peral et al. 2018; Supplementary Table S1). This can be compensated with a higher level of consistency at the individual-level.

### Behavioral traits affecting the production parameters

Personality dimensions, such as aggression, exploration, and boldness, were found to have important fitness consequences in many different species (Smith and Blumstein 2008; Modlmeier and Foitzik 2011; Modlmeier et al. 2012). Based on our results, it seems that this effect can be highly influenced by the different habitat types selective for various behavioral traits regarding their effect on the colony fitness parameter like reproduction and growth. Moreover, our results also showed that some behavioral traits, such as exploration, can have strong fitness consequences in ants and can be selected in a similar way by different habitats and also at different levels of organization (individual and colony). This trait in both habitats strongly affected total production and the production of new workers, so it may play an important role in discovering new sources of carbohydrates, as workers require mainly a carbohydrate diet for energy (Dussutour and Simpson 2009). Generally, it is not well known how the role of collective exploration determines colony success (Wright et al. 2019). Although exploratory animals have a higher chance of discovering food sources, they also take higher risks and have higher metabolic rates (Careau et al. 2008).

On the colony-level higher meanderness and variability of aggression, and lower variability of meanderness results in higher worker and total production. This is partially in line with the findings on *Temnothorax* ants, where higher variability in aggression and exploration was found to enhance productivity (Modlmeier and Foitzik 2011; Modlmeier et al. 2012). These results also suggested that higher behavioral variation can be closely related to higher colony success (see Modlmeier et al. 2012), as colonies with behavioral variation among workers can react faster and more appropriately to changing colony needs and should show more efficient task allocation (Myerscough and Oldroyd 2004). The differences may be related to the age of workers, as we tested only foragers, which, being the oldest individuals, have the most experience, which can enhance aggression (Van Wilgenburg et al. 2010). Moreover, foragers also show higher exploratory tendencies, making it possible to acquire important information about the state of the colony and its environment, for example, the location of food sources (Herbers and Choiniere 1996). Also, higher nest displacement efficiency and its lower variability affected positively worker and total production. This suggests that decision-making strategies, besides involving collective personality (Modlmeier et al. 2014), can have very important fitness consequences at the colony-level, mostly in habitats with high nest densities involving intense competition for new nesting sites. Unfortunately, until now, no work has quantified the among-colony variation of any cognitive trait (Wright et al. 2019), nor its effect on colony productivity.

On the individual-level, different habitat types can change the effect of different behavioral traits on productivity. It seems that in seminatural meadows (Lenda et al. 2013; Grześ et al. 2018; Trigos-Peral et al. 2018), where higher nest densities occur, selection acts mostly for the aggression of workers that can result in increased overall productivity (see also Modlmeier and Foitzik 2011). The higher nest density can also lead to frequent encounters with foragers of other colonies (Hölldobler and Wilson 1990), so a more targeted path (lower meandering) when gathering resources can also enhance productivity. On the other hand, in invaded meadows, the activity of workers had a positive effect on total production and the production of new workers (Table 6), suggesting higher selection pressure on this behavioral trait. It seems that workers in the invaded habitats need to cover larger distances to ensure their food intake, while the importance of aggression is less accentuated. These findings also underpin the suggestions of Modlmeier and Foitzik (2011) that higher productivity and greater variability in aggression could be the result of good habitat quality. Based on the former, it seems that more natural habitats favor behavioral traits associated with aggression and the directionality of the individual movement, while more disturbed habitats favor higher activity. An important component of this axis is increased aggression, which can be predictive of success in a wide variety of contexts, but also has costs in others; therefore, it might be under balancing selection in many insect systems (Wright et al. 2019).

## CONCLUSIONS

Our work implies that habitat and colony characteristics can highly influence the behavioral traits of an ant colony, which in turn can have important fitness consequences. Colony size and nest density are the most important factors shaping variation of ant behavior, whereas the effects of behavioral traits on colony fitness seem to highly depend on the social level (colony or individual). Our results also highlight the need to perform complex studies on various levels of behavioral variation of social insects (among- and within-individual/colony variation and also among worker/colony residual variation), as only by integrating all these elements we will be able to fully understand the effects shaping behavior and productivity parameters. Moreover, by clarifying the details of the ecology of the collective behaviors of social-insect colonies and their adaptive role in certain environments we will be able to grasp the factors determining their fitness under particular habitat circumstances.

## Supplementary Material

araa112_suppl_Supplementary_Code

araa112_suppl_Supplementary_Table
