# Supplementary material for: Habitat features and colony characteristics influencing ant personality and its fitness consequences
Source: Behav Ecol. 2020 Nov 10;32(1):124–37. doi: 10.1093/beheco/araa112 (PMC7937185; doi:10.1093/beheco/araa112)
Supplement: araa112_suppl_Supplementary_Table [file araa112_suppl_supplementary_table.docx]

**Table S1. Repeatability estimates for the studied behavioural traits separately for the two habitat types on colony and on individual level of *Myrmica rubra* ants. Repeatability values (R) and 95 % confidence intervals (CI) are shown. Significance (*P*) estimates are based on randomization tests.**

| Behavioral traits | Semi-natural meadows  R (95% CrI) | | Solidago meadows  R (95% CrI) | |
| --- | --- | --- | --- | --- |
|  | **Colony level**  *N* = 36 | **Individual level**  *N* = 366 | **Colony level**  *N* = 33 | **Individual level**  *N* = 315 |
| Activity | **0.27 (0.081 – 0.449)** | **0.28 (0.173 – 0.406)** | **0.13(0 – 0.286)** | **0.38 (0.256 – 0.516)** |
| Aggression | **0.16 (0.032 – 0.291)** | **0.26 (0.151 – 0.386)** | **0.1 (0 – 0.235)** | **0.27 (0.157 – 0.402)** |
| Exploration | **0.23 (0.072 – 0.383)** | **0.16 (0.066 – 0.272)** | **0.13 (0.008 – 0.285)** | **0.29 (0.156 – 0.415)** |
| Meandering | **0.37 (0.14 – 0.558)** | **0.21 (0.116 – 0.337)** | **0.11 (0 – 0.235)** | **0.37 (0.236 – 0.491)** |
| Nest disp. | **0.36 (0 – 0.683)** |  | **0.42 (0 – 0.711)** |  |

Effects strongly supported by the model (95% CI not overlapping) are in bold font.
